# Long working hours, socioeconomic status, and the risk of incident type 2 diabetes: a meta-analysis of published and unpublished data from 222 120 individuals

**DOI:** 10.1016/S2213-8587(14)70178-0

**Published:** 2015-01

**Authors:** Mika Kivimäki, Marianna Virtanen, Ichiro Kawachi, Solja T Nyberg, Lars Alfredsson, G David Batty, Jakob B Bjorner, Marianne Borritz, Eric J Brunner, Hermann Burr, Nico Dragano, Jane E Ferrie, Eleonor I Fransson, Mark Hamer, Katriina Heikkilä, Anders Knutsson, Markku Koskenvuo, Ida E H Madsen, Martin L Nielsen, Maria Nordin, Tuula Oksanen, Jan H Pejtersen, Jaana Pentti, Reiner Rugulies, Paula Salo, Johannes Siegrist, Andrew Steptoe, Sakari Suominen, Töres Theorell, Jussi Vahtera, Peter J M Westerholm, Hugo Westerlund, Archana Singh-Manoux, Markus Jokela

**Affiliations:** a Department of Epidemiology and Public Health, University College London, London, UK; b Hjelt Institute, Medical Faculty, University of Helsinki, Helsinki, Finland; c Finnish Institute of Occupational Health, Helsinki, Tampere, and Turku, Finland; d Harvard School of Public Health, Department of Society, Human Development and Health, Boston, MA, USA; e Centre for Occupational and Environmental Medicine, Stockholm County Council, Sweden; f Institute of Environmental Medicine, Karolinska Institutet, Stockholm, Sweden; g Centre for Cognitive Ageing and Cognitive Epidemiology, University of Edinburgh, Edinburgh, UK; h Alzheimer Scotland Dementia Research Centre, University of Edinburgh, Edinburgh, UK; i National Research Centre for the Working Environment, Copenhagen, Denmark; j Department of Occupational and Environmental Medicine, Bispebjerg University Hospital, Copenhagen, Denmark; k Federal Institute for Occupational Safety and Health, Berlin, Germany; l Institute for Medical Sociology, Medical Faculty, University of Düsseldorf, Düsseldorf, Germany; m School of Social and Community Medicine, University of Bristol, Bristol, UK; n School of Health Sciences, Jönköping University, Jönköping, Sweden; o Stress Research Institute, Stockholm University, Stockholm, Sweden; p School of Medicine, University of Tampere, Tampere, Finland; q Department of Health Sciences, Mid Sweden University, Sundsvall, Sweden; r Department of Public Health, University of Helsinki, Helsinki, Finland; s Department of Psychology, Umeå University, Umeå, Sweden; t Danish National Centre for Social Research, Copenhagen, Denmark; u Department of Public Health and Department of Psychology, University of Copenhagen, Copenhagen, Denmark; v Department of Psychology, University of Turku, Turku, Finland; w Folkhälsan Research Center, Helsinki, Finland; x Nordic School of Public Health, Göteborg, Sweden; y Department of Public Health, University of Turku, Turku, Finland; z Turku University Hospital, Turku, Finland; aa Occupational and Environmental Medicine, Uppsala University, Uppsala, Sweden; ab Inserm U1018, Centre for Research in Epidemiology and Population Health, Villejuif, France; ac Institute of Behavioral Sciences, University of Helsinki, Helsinki, Finland

## Abstract

**Background:**

Working long hours might have adverse health effects, but whether this is true for all socioeconomic status groups is unclear. In this meta-analysis stratified by socioeconomic status, we investigated the role of long working hours as a risk factor for type 2 diabetes.

**Methods:**

We identified four published studies through a systematic literature search of PubMed and Embase up to April 30, 2014. Study inclusion criteria were English-language publication; prospective design (cohort study); investigation of the effect of working hours or overtime work; incident diabetes as an outcome; and relative risks, odds ratios, or hazard ratios (HRs) with 95% CIs, or sufficient information to calculate these estimates. Additionally, we used unpublished individual-level data from 19 cohort studies from the Individual-Participant-Data Meta-analysis in Working-Populations Consortium and international open-access data archives. Effect estimates from published and unpublished data from 222 120 men and women from the USA, Europe, Japan, and Australia were pooled with random-effects meta-analysis.

**Findings:**

During 1·7 million person-years at risk, 4963 individuals developed diabetes (incidence 29 per 10 000 person-years). The minimally adjusted summary risk ratio for long (≥55 h per week) compared with standard working hours (35–40 h) was 1·07 (95% CI 0·89–1·27, difference in incidence three cases per 10 000 person-years) with significant heterogeneity in study-specific estimates (*I*^2^=53%, p=0·0016). In an analysis stratified by socioeconomic status, the association between long working hours and diabetes was evident in the low socioeconomic status group (risk ratio 1·29, 95% CI 1·06–1·57, difference in incidence 13 per 10 000 person-years, *I*^2^=0%, p=0·4662), but was null in the high socioeconomic status group (1·00, 95% CI 0·80–1·25, incidence difference zero per 10 000 person-years, *I*^2^=15%, p=0·2464). The association in the low socioeconomic status group was robust to adjustment for age, sex, obesity, and physical activity, and remained after exclusion of shift workers.

**Interpretation:**

In this meta-analysis, the link between longer working hours and type 2 diabetes was apparent only in individuals in the low socioeconomic status groups.

**Funding:**

Medical Research Council, European Union New and Emerging Risks in Occupational Safety and Health research programme, Finnish Work Environment Fund, Swedish Research Council for Working Life and Social Research, German Social Accident Insurance, Danish National Research Centre for the Working Environment, Academy of Finland, Ministry of Social Affairs and Employment (Netherlands), Economic and Social Research Council, US National Institutes of Health, and British Heart Foundation.

## Introduction

Type 2 diabetes, characterised by hyperglycaemia and insulin resistance or insulin insufficiency, causes substantial disease burden.^1^ Globally, more than 285 million people have type 2 diabetes, and its prevalence is predicted to increase to 439 million by 2030.^2^ The findings from prospective cohort studies show that working long hours is associated with factors that contribute to diabetes, such as unhealthy lifestyle, work stress, sleep disturbances, and depressive symptoms.^3^ Working long hours is also associated with an increased risk of cardiovascular disease,^4^, ^5^ which is one of the complications of type 2 diabetes. However, the direct association between long working hours and incident type 2 diabetes has been assessed in only a few studies.^6^, ^7^, ^8^, ^9^

In some occupations, long working hours can increase an individual's exposure to health hazards over time—eg, physical inactivity or psychosocial stress.^3^, ^5^ For example, recent findings showed that the adverse effect of long working hours on cardiovascular disease is more marked in individuals who have manual and other low socioeconomic status occupations than in those who have high socioeconomic status jobs.^5^ Findings from two Japanese studies lend indirect support that this might also be the case in relation to incident type 2 diabetes.^6^, ^7^ In a study of industrial employees who were mostly manual workers, the risk of incident type 2 diabetes was increased in those working long hours,^6^ whereas in another study of only office employees there was no increase in the risk of diabetes.^7^ These two studies also differed in several other respects, including the setting, measurement of working hours, and incident diabetes. Therefore, whether the discrepant results are due to factors other than socioeconomic status is not known.

Using a socioeconomic status-stratified meta-analysis, we tested the hypothesis that an association between working hours and type 2 diabetes would be stronger in individuals with a low socioeconomic status than in those with a high socioeconomic status.

## Methods

### Search strategy and selection criteria

We undertook a meta-analysis, in accordance with the PRISMA guidelines,^10^ of published studies identified in a systematic review and supplemented this with unpublished individual-level data to assess the status of long working hours as a risk factor for type 2 diabetes. We did a systematic search of the literature up to April 30, 2014, in PubMed and Embase, using the following terms with no restrictions: for exposure we used “(working and hours) or (overtime) or (overtime and work)”, and for outcome we used “(diabetes) or (prediabetes) or (diabetes and mellitus) or (impaired and fasting and glucose) or (glucose) or (impaired and glucose and tolerance) or (borderline and diabetes) or (blood and glucose) or (haemoglobin and A) or (glycosylated)”. We also searched the reference lists of all relevant publications that were identified. Additionally, we used the Institute of Scientific Information Web of Science to retrieve all the study reports citing the studies identified in the search.

MKi and MV independently reviewed the titles and abstracts of the studies identified by the literature search to identify potentially relevant studies using a broad range of criteria for exposure (work hours) and outcome (diabetes) to further assess eligibility. Selected full articles were reviewed by MKi and MV to ascertain whether they met the inclusion criteria.

We included studies that met the following criteria: English language publication; prospective design (cohort study) with individual-level exposure and outcome data; investigation of the effect of working hours or overtime work; incident diabetes as the outcome; and relative risks, odds ratios, or hazard ratios (HRs) with 95% CIs reported or sufficient information provided to calculate these estimates.

### Unpublished individual-participant data

We gathered individual-level data by searching collections of the Inter-University Consortium for Political and Social Research (ICPSR) and the UK Data Service to identify eligible large-scale cohort studies for which data were publicly available. Eleven cohort studies were identified.^11^, ^12^, ^13^, ^14^, ^15^, ^16^, ^17^, ^18^, ^19^, ^20^, ^21^ Furthermore, we included individual-level data from eight relevant cohort studies from the Individual-Participant-Data Meta-analysis in Working Populations (IPD-Work) Consortium.^22^, ^23^, ^24^, ^25^, ^26^, ^27^, ^28^, ^29^, ^30^

Each constituent study from the IPD-Work Consortium, ICPSR, and the UK Data Service was approved by the relevant local or national ethics committees, and all participants gave informed consent to take part.

In all these datasets, we defined long working hours as 55 h or more of work per week and the reference category as 35–40 h of work per week.^4^ As previously,^22^ we defined socioeconomic status on the basis of occupational title categorised into high (eg, managers or directors), intermediate (eg, clerical, sales, or skilled non-manual), and low (eg, manual workers). Information about occupation was not available in one study and socioeconomic status was defined on the basis of a participant's self-reported highest educational qualification.^28^ Covariates were age, sex, smoking, BMI, physical activity, and alcohol consumption. Additionally, we assessed shift work, a known risk factor for type 2 diabetes.^31^, ^32^ Incident type 2 diabetes was ascertained with blood testing,^33^ or records from health registers or self-reports (appendix).

### Statistical analysis

The open-access studies had self-reported incident diabetes without a precise date so we used logistic regression to calculate odds ratios and 95% CIs for the association between working hours and incident type 2 diabetes in each study. In the IPD-Work studies,^22^, ^23^, ^24^, ^25^, ^26^, ^27^, ^28^, ^29^, ^30^ the date of diagnosis was available and the proportional hazards assumption was not violated, so we used Cox proportional hazards models to generate HRs and 95% CIs. Because the incidence of type 2 diabetes was low, odds ratios were judged to be close approximations of relative risk. They were therefore combined with HRs, resulting in a common estimate of effect size (appendix). Absolute differences in incidences of diabetes were calculated by first ascertaining the incidence in the reference group of participants working normal hours (35–40 h) using sample-size weighted mean. This base incidence was then multiplied by the meta-analysis HR estimate for long working hours to calculate absolute incidence difference associated with long working hours.

In published studies, the associations were reported as HRs or odds ratios and regarded as relative risk ratios in our analysis. From these studies, we obtained categories for long working hours and normal working hours (reference) that were closest to those used in the individual-participant datasets (≥55 h *vs* 35–40 h worked per week). Conventional meta-analytical methods were used to combine results from the individual-level datasets and estimates from studies identified in the systematic literature search.

The base model in all analyses was minimally adjusted, including only age and sex in addition to working hours. To assess the effect of known diabetes risk factors on the association, we did multivariable (maximally adjusted) analyses. In the individual-participant datasets, these models were adjusted for age, sex, socioeconomic status (except in socioeconomic status-specific analyses), smoking, alcohol consumption, BMI, and physical activity.

We assessed heterogeneity of the study-specific estimates using the *I*^2^ statistic (a higher value indicates a greater degree of heterogeneity) and provided summary estimates of the random-effects analysis.^34^ To assess the hypothesised effect modification by socioeconomic status, we stratified the study-specific analyses by socioeconomic status and pooled the study-specific estimates separately for low, intermediate, and high socioeconomic status groups. We used *z* statistics to ascertain whether the association between long working hours and type 2 diabetes was stronger in the low socioeconomic status group than in the high socioeconomic status group (appendix). Prespecified subgroup differences in the working hours–diabetes association according to the method of diabetes ascertainment (self-reported *vs* national health registers or blood-based test), length of follow-up (<5 years *vs* >5 years), study location (the USA, Europe, Japan, or Australia), sex (female *vs* male), age group (>50 year *vs* <50 years), and obesity (BMI <30 kg/m^2^ *vs* >30 kg/m^2^) status were also assessed with *z* statistics. Additionally, we repeated the analysis after excluding the first 3 years of follow-up to reduce reverse causation bias.

Study-specific data from the IPD-Work studies was analysed using SAS (version 9.2). Data analysis of ICPSR and the UK Data Service studies was done using Stata (version 11.2), which was also used to compute the results of all meta-analyses.

### Role of the funding source

The study funders did not contribute to the study design and had no role in data gathering, analysis, or interpretation, or the writing of the report. MKi, STN, and MJ had full access to all unpublished individual-level data in this meta-analysis (with the exception of data from COPSOQ-I,^26^ COPSOQ-II,^27^ and DWECS,^30^ which were analysed by IEHM) and take final responsibility for the decision to submit for publication.

## Results

**Figure 1:**
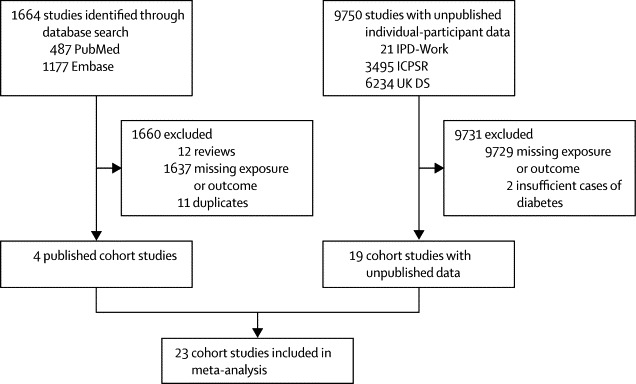
Study selection IPD-Work=Individual-Participant-Data Meta-analysis in Working Populations Consortium. ICPSR=Inter-University Consortium for Political and Social Research. UK DS=UK Data Service.

We identified 1664 published studies through our literature search (figure 1). Of these, 1660 studies were not eligible and were excluded, and four cohort studies met the inclusion criteria for the meta-analysis. These studies were from Japan,^6^, ^7^ the USA,^8^ and Sweden (table).^9^ Of the unpublished cohort studies from ICPSR, the UK Data Service, and IPD-Work, 19 studies from the USA, the UK, Finland, Sweden, Denmark, and Australia were eligible for inclusion in the meta-analysis.^11^, ^12^, ^13^, ^14^, ^15^, ^16^, ^17^, ^18^, ^19^, ^20^, ^21^, ^22^, ^23^, ^24^, ^25^, ^26^, ^27^, ^28^, ^29^, ^30^ The published and unpublished data represented 222 120 men and women with 4963 incident cases of diabetes over 1·7 million person-years of follow-up (mean 7·6 years).

**Table:** Characteristics of participants from unpublished and published studies

		Year*	**Country**	**Number of participants**	**Number (%) of women**	**Mean age at baseline (years; SD)**	**Number (%) of participants with long working hours**	**Number of diabetes cases**	**Method of ascertainment**	**Mean follow-up (years; SD)**	**Person-years**	**Incidence per 10 000 person-years**†	**Socioeconomic status stratification**‡
Unpublished studies													
	ACL^11^	1986	USA	1493	787 (53%)	44·5 (13·7)	178 (12%)	163	Self-report	12·6 (4·2)	18 785	87†	Yes
	Alameda^12^	1973	USA	1461	484 (33%)	44·1 (9·6)	148 (10%)	127	Self-report	25·5 (0·4)	37 278	34†	Yes
	BCS1970^19^	2004	UK	6447	3100 (48%)	33·8 (1·8)	369 (6%)	51	Self-report	4·0 (0·2)	25 483	20†	Yes
	BHPS^17^	1991	UK	15 238	7836 (51%)	34·1 (11·1)	1165 (8%)	267	Self-report	6·4 (4·0)	96 877	28†	Yes
	HILDA^21^	2005	AUS	4856	2322 (48%)	41·4 (12·6)	542 (11%)	77	Self-report	4·0 (0·1)	19 443	40†	Yes
	MIDUS^14^	1995	USA	2954	1540 (52%)	44·9 (8·9)	394 (13%)	188	Self-report	8·9 (0·4)	26 355	71†	Yes
	NCDS^18^	2000	UK	7678	3697 (48%)	42·0 (0·0)	594 (8%)	207	Self-report	8·3 (0·4)	63 555	33†	Yes
	NHANES-I^13^	1982	USA	4976	2835 (57%)	48·9 (10·6)	484 (10%)	228	Self-report	9·1 (1·5)	45 153	50†	Yes
	UndSoc^20^	2009	UK	10 969	6183 (56%)	42·5 (12·5)	559 (4%)	259	Self-report	2·9 (0·3)	31 809	81†	Yes
	WLSG^15^	1992	USA	5524	2907 (53%)	54·1 (0·5)	744 (13%)	493	Self-report	11·2 (0·3)	61 684	80†	Yes
	WLSS^16^	1993	USA	2569	1376 (54%)	52·6 (7·0)	355 (14%)	222	Self-report	11·2 (0·5)	28 900	77†	Yes
	FPS^29^	2000	Finland	43 600	35 128 (81%)	44·5 (9·4)	1387 (3%)	1107	Health register	9·6 (1·1)	418 093	26	Yes
	HeSSup^28^	1998	Finland	15 931	8856 (56%)	39·5 (10·2)	1386 (9%)	128	Health register	6·9 (0·4)	110 670	12	Yes
	Whitehall II^23^	1985	UK	7263	2197 (30%)	49·0 (5·8)	726 (10%)	579	Blood test	12·6 (3·3)	91 670	63	Yes
	WOLF N^25^	1996	Sweden	4576	759 (17%)	43·9 (10·3)	52 (1%)	49	Blood test	11·6 (1·2)	52 967	9	Yes
	WOLF S^24^	1992	Sweden	5497	2372 (43%)	41·4 (11·0)	227 (4%)	80	Blood test	14·4 (1·9)	79 425	10	Yes
	COPSOQ-I^26^	1997	Denmark	1798	870 (48%)	40·6 (10·6)	109 (6%)	47	Health register	12·6 (2·0)	22 621	21	Yes
	COPSOQ-II^27^	1998	Denmark	3320	1747 (53%)	42·6 (10·2)	175 (5%)	21	Health register	5·9 (0·6)	19 709	11	Yes
	DWECS^30^	2000	Denmark	5505	2573 (47%)	41·8 (11·0)	439 (8%)	68	Health register	9·8 (1·4)	53 693	13	Yes
Published studies													
	Kawakami^6^	1999	Japan	2194	0	37·1§	351 (16%)	34	Blood test	8·0§	17 451	19	No
	Nakanishi^7^	2001	Japan	1266	0	46·7§	174 (14%)	54	Blood test	5·0§	6330	85	No
	Kroenke^8^	2007	USA	62 574	62 574 (100%)	38·8§	1482 (2%)	365	Self report	5·6§	351 363	10	No
	Eriksson^9^	2013	Sweden	4431	2707 (61%)	46·7§	2997 (68%)	149	Blood test	10·1§	44 865	33	No
Total				222 120	153 067 (69%)	43·3§	15 047 (7%)	4963		7·6§	1 724 179	28·8	

* Baseline year for unpublished studies and publication year for published studies.

† Incidence calculated by use of total follow-up (incidence dates were not available); in the other studies, each participant was followed up from the date of their baseline assessment to the earliest incident of diabetes, death, or the end of follow-up.

‡ Included in socioeconomic-status-stratified analyses.

§ SD was not available for the published studies.

The definitions of long and normal working hours varied between the published studies,^6^, ^7^, ^8^, ^9^ whereas the definition was uniform in the unpublished studies (≥55 h *vs* 35–40 h worked per week).^11^, ^12^, ^13^, ^14^, ^15^, ^16^, ^17^, ^18^, ^19^, ^20^, ^21^, ^22^, ^23^, ^24^, ^25^, ^26^, ^27^, ^28^, ^29^, ^30^ In the Japanese study of industrial employees, long hours entailed working for more than 53 h per week,^6^ the corresponding threshold was longer than 55 h per week in the Japanese study of male office workers^7^ and more than 60 h per week in a US study of female nurses.^8^ In a study of the Swedish working population, long working hours were assessed with a question about whether the participants worked overtime.^9^ The proportion of employees working long hours ranged from 1·1% to 13·8% in unpublished studies^11^, ^12^, ^13^, ^14^, ^15^, ^16^, ^17^, ^18^, ^19^, ^20^, ^21^, ^22^, ^23^, ^24^, ^25^, ^26^, ^27^, ^28^, ^29^, ^30^ and from 2·5% to 67·6% in published studies.^6^, ^7^, ^8^, ^9^

In published studies, an oral glucose tolerance test was used to ascertain diabetes in one study,^9^ fasting glucose was used to define diabetes in two studies,^6^, ^7^ and diabetes was self-reported in one study.^8^ In the unpublished studies, the oral glucose tolerance test was used to ascertain diabetes in one study,^23^ electronic patient records in seven studies,^23^, ^24^, ^25^, ^26^, ^27^, ^28^, ^29^, ^30^ and self-report in 11 studies.^11^, ^12^, ^13^, ^14^, ^15^, ^16^, ^17^, ^18^, ^19^, ^20^, ^21^ The mean incidence of diabetes per 10 000 person-years was 28·8 (range 9–87; table). 70·1% of the variation in diabetes incidence between studies was due to differences in age and sex distributions of the study population, length of follow-up, and the method used to define incident diabetes.

**Figure 2:**
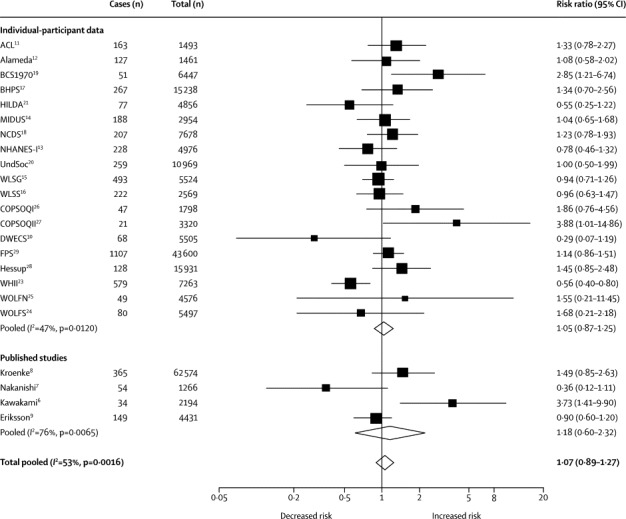
Random-effects meta-analysis of unpublished and published studies of minimally adjusted association between working long hours and incident type 2 diabetes

Figure 2 shows the results of the meta-analyses for both unpublished (n=151 655)^11^, ^12^, ^13^, ^14^, ^15^, ^16^, ^17^, ^18^, ^19^, ^20^, ^21^, ^22^, ^23^, ^24^, ^25^, ^26^, ^27^, ^28^, ^29^, ^30^ and published studies (n=70 465).^6^, ^7^, ^8^, ^9^ In the minimally adjusted model, the summary risk ratio for incident type 2 diabetes was 1·07 (95% CI 0·89–1·27, difference in incidence three cases per 10 000 person-years) for people working long hours compared with those working standard hours (*I*^2^=53%, p=0·0016; figure 2). The relative risk for people working long hours compared with those working normal hours was similar in unpublished and published studies. Heterogeneity in the study-specific estimates was significant in both unpublished and published studies (*I*^2^=47%, p=0·0120, and *I*^2^=76%, p=0·0065, respectively; figure 2).

**Figure 3:**
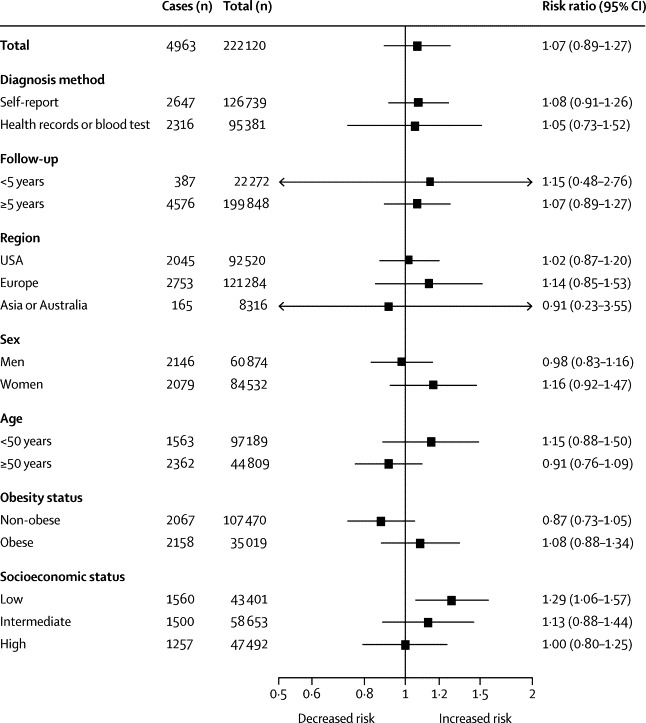
Association between working long hours and incident type 2 diabetes by subgroup in individual-participant datasets from the Individual-Participant-Data Meta-analysis in Working Populations Consortium, Inter-University Consortium for Political and Social Research, and the UK Data Service resources

Figure 3 shows the results from additional analyses that were done to identify sources of heterogeneity between the studies (some data were missing in these subgroup analyses). The association between long working hours and diabetes was stronger in the low socioeconomic status group (risk ratio 1·29, 95% CI 1·06–1·57, difference in incidence 13 cases per 10 000 person-years) than in the high socioeconomic status group (1·00, 0·80–1·25, zero per 10 000 person-years; figure 3; p_interaction_=0·0965, p_one-tailed_=0·0483). In this stratified analysis, there was no longer heterogeneity in study-specific estimates (*I*^2^=0%, p=0·6981 in the low socioeconomic status group; *I*^2^=10%, p=0·3377 in the high socioeconomic status group). In the intermediate socioeconomic status group, the risk ratio suggested an association between long working hours and diabetes according to the grade of socioeconomic status (1·13, 0·88–1·44, four per 10 000 person-years). The summary estimate did not vary according to the method of diagnosing diabetes (p_interaction_=0·9158), length of follow-up (p=0·8704), study location (multiple comparisons, all p>0·5275), sex (p=0·9158), age group (p=0·1521), or obesity status (p=0·1340); *z* statistics are reported in the appendix.

**Figure 4:**
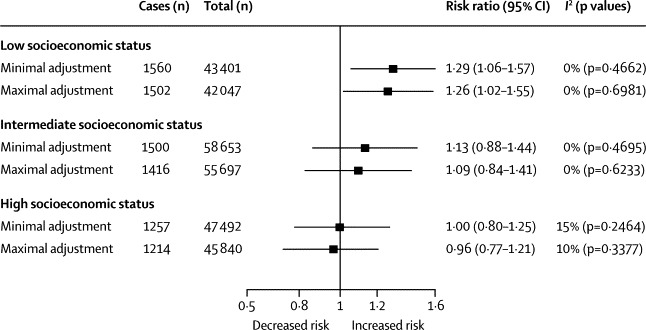
Minimally and maximally adjusted risk ratios for the association between working long hours and risk of incident type 2 diabetes by socioeconomic status

Figure 4 shows the serially adjusted meta-analyses of the association between long working hours and incident diabetes stratified by socioeconomic status. In the multivariable-adjusted analyses, the relative risk of incident diabetes for long working hours compared with standard working hours in the low socioeconomic status group was 1·26 (95% CI 1·02–1·56, difference in incidence 12 cases per 10 000 person-years; figure 4).

In further sensitivity analyses (see appendix for details and results), we did not find evidence of reverse causation bias. In low socioeconomic status groups, the summary estimate was little changed after the exclusion of the first 3 years of follow-up (appendix). Furthermore, there was no evidence of an increased diabetes risk in part-time workers (<35 h per week; appendix). To assess whether the association between long working hours and type 2 diabetes is due to shift work, we repeated the analysis with shift workers^17^, ^19^, ^21^, ^23^, ^28^, ^29^ excluded from the sample; the association remained unchanged.

## Discussion

The results of this meta-analysis suggest that working long hours does not uniformly increase the risk of developing type 2 diabetes. The findings also lend support to our hypothesis of a stronger association in low socioeconomic groups: individuals who worked 55 h or more per week had an almost 30% increased risk of developing diabetes than did those who worked 35–40 h per week. This association was robust to adjustments for age, sex, BMI, and lifestyle factors, such as leisure-time physical activity, smoking, and alcohol consumption. It also remained after shift workers were excluded from the analysis.

Our quantitative review complements previous narrative reviews that suggest that working long hours might be associated with increased morbidity.^3^, ^35^ We are aware of only one previous meta-analysis of long working hours and incident type 2 diabetes. It was based on three cohort studies with a total of 453 incident diabetes cases; it did not report socioeconomic-status-specific results, and found no overall association between working hours and diabetes.^36^ With a total of 222 120 participants and 4963 incident diabetes cases, our meta-analysis—which includes data from the three published studies that were included in the previous meta-analysis—is the largest study so far.

Results from single studies of long working hours and incident type 2 diabetes have been mixed, with one study supporting an association^6^ and three studies finding no association.^7^, ^8^, ^9^ The only published study that suggested increased diabetes risk in individuals working long hours focused on industrial employees who were mostly in manual occupations.^6^ By contrast, in the three investigations with null findings the study population comprised employees of both high and low socioeconomic status^8^, ^9^ or only office workers.^6^ We noted a large heterogeneity in study-specific estimates in analyses combining socioeconomic status groups, but no heterogeneity when analyses were stratified by socioeconomic status. Our meta-analysis suggests that failure to take into account the socioeconomic status-specific pattern largely explains discrepancies in previous published studies.

We found no evidence that the association between working hours and diabetes differs between men and women, old and young, obese and non-obese participants, or by regions. Studies using self-reports to ascertain diabetes are subject to recall bias and cannot take into account undiagnosed cases. However, we noted a similar association between working hours and diabetes in studies with different methods to define diabetes, including blood-based tests that capture undiagnosed diabetes and avoid recall and reporting bias.

We defined working long hours as 55 h or more per week, a commonly used definition in previous studies,^4^ and the reference category as 35–40 h of work per week, which is the standard working week for most people. This categorisation provides an unambiguous definition for long working hours and allowed us to estimate increased risk in relative and absolute terms with standard working hours as the reference. We did not treat working hours as a continuous variable because the interpretation of results is less straightforward and none of the published studies provided relevant data.^6^, ^7^, ^8^, ^9^

Reverse-causation bias can mask the association between long hours and type 2 diabetes if individuals with undiagnosed diabetes or advanced prediabetes opt to work shorter hours because of tiredness associated with these illnesses. To explore this possibility, we excluded the first 3 years of follow-up from the analysis and found no evidence for reverse causation. Furthermore, individuals working reduced hours (<35 h per week) did not have higher diabetes risk than did those working normal hours.

The mechanisms underlying the association between long working hours and diabetes in the low socioeconomic status group are yet unknown. There are at least three different ways in which long hours could be hazardous. First, working long hours might be a marker of other risk factors, and once these are controlled for, it is not hazardous. According to this explanation, the residual excess risk in workers with a low socioeconomic status might be due to confounding by unmeasured confounders, such as low pay and financial constraints, with long working hours in these workers being simply a marker of personal hardships. By contrast, for many workers with a high socioeconomic status who do not have such hardships, working long hours is not difficult and is often voluntary to make more money or achieve important goals. Hence, for these workers, long working hours might not be hazardous to health.

Second, working long hours might be causally related to health problems because it displaces other health-restorative behaviours, particularly sleep, time to rest, and time to engage in physical activity and social interactions. This is a causal explanation, but one that assumes a causal effect of other, more proximal risk factors. In our meta-analysis, physical activity, smoking, alcohol consumption, and obesity did not explain the association between long working hours and diabetes. Future research should focus on other potential mediating factors. For example, sleep deprivation and sleep fragmentation are associated with multiple mechanisms that increase the risk of type 2 diabetes, including decreased β-cell function and increased insulin resistance.^37^, ^38^, ^39^ Similarly, those with chronic stress at work, chronic anger, or who are socially isolated are at an increased risk of developing metabolic syndrome or diabetes.^32^, ^40^

Third, there is a possibility that long work hours are hazardous, without the other indirect mechanisms. This effect could be due to deleterious consequences of working long working hours on personal growth and fulfilment, and happiness, but the link to the risk of physical disease seems tenuous. If being at work for more than 55 h per week is bad for metabolic health, then we ought to see an effect also in workers with a high socioeconomic status. The fact that we do not see it suggests that the association is driven by confounding or indirect effects on other risk factors.

Our results need to be considered in view of study limitations. We used observational data and so cannot eliminate confounding or confirm a causal relation between working hours and disease risk. Our systematic literature search covered studies published only in English and, although the published and unpublished data in our meta-analysis covered the USA, Japan, Australia, and several countries in Europe, not all of these studies were population based. Thus, the generalisability of our findings remains uncertain. Also, we measured working hours based on a single assessment that might not represent long-term exposure. In further studies, investigators should use repeat measurements to characterise exposure to long working hours. By the same token, confounding factors are also best assessed using repeat measurements.

In conclusion, our findings add to the understanding of the adverse health effects of long working hours by suggesting that working 55 h or more per week is associated with an increased risk of type 2 diabetes, but only in individuals from low socioeconomic status groups.
