# Supplementary material for: Long working hours, socioeconomic status, and the risk of incident type 2 diabetes: a meta-analysis of published and unpublished data from 222 120 individuals
Source: Lancet Diabetes Endocrinol. 2015 Jan;3(1):27–34. doi: 10.1016/S2213-8587(14)70178-0 (PMC4286814; doi:10.1016/S2213-8587(14)70178-0)
Supplement: Supplementary appendix [file mmc1.pdf]

THE LANCET Diabetes & Endocrinology

Supplementary appendix

This appendix formed part of the original submission and has been peer reviewed. We post it as supplied by the authors.

Supplement to: Kivimäki M, Virtanen M, Kawachi I, et al. Long working hours, socioeconomic status, and the risk of incident type 2 diabetes: a meta-analysis of published and unpublished data from 222 120 individuals. *Lancet Diabetes Endocrinol* 2014; published online Sept 25. http://dx.doi.org/10.1016/S2213-8587(14)70178-0.

WEB APPENDIX

Kivimäki M, Virtanen M, Kawachi I et al. Long working hours, socioeconomic status, and the risk of incident type 2 diabetes: Meta-analysis of published and unpublished data from 222,120 individuals. *Lancet: Diabetes & Endocrinology* 2014

Individual-participant datasets

Individual-level data from collections of the Inter-University Consortium for Political and Social Research (ICPSR; http://www.icpsr.umich.edu/icpsrweb/ICPSR/) and the UK Data Service (http://ukdataservice.ac.uk/): the American's Changing Lives (ACL) study;[1] the Alameda County study, US;[2] the National Health and Nutrition Survey I (NHANES-I);[3] the Midlife in the United States (MIDUS);[4] the Wisconsin Longitudinal Study, graduates (WLSG)[5] and siblings (WLSS);[6] the British Household Panel Survey (BHPS);[7] the British 1958 Birth Cohort (also known as the 1958 National Child Development Study, NCDS);[8] the British Birth Cohort 1970;[9] the Understanding Society cohort;[10] and the Household, Income and Labour Dynamics in Australia survey (HILDA).[11]

Individual-level data from 8 European prospective cohort studies participating in the Individual-Participant-Data Meta-analysis in Working Populations (IPD-Work) Consortium:[12] the Whitehall II study, UK[13] Work, Lipids and Fibrinogen study (WOLF), Stockholm,[14] WOLF, Norrland, Sweden,[15] COPSOQ-I,[16] COPSOQ-II, Denmark,[17] Health and Social Support study (HeSSup), Finland,[18] Finnish Public Sector Study (FPS), Finland,[19] and the Danish Work Environment Cohort Study (DWECS), Denmark.[20]

Measurement of socioeconomic status, covariates and type 2 diabetes executive

In the open-access datasets,[1-11] SES was determined on the basis of Census occupational classification groups in the American cohorts[1-6] ("High"= professional, executive, technical; "Intermediate"= clerical, sales, administration; "Low" = craft, operatives, service, labour, farmer), the Registrar General's social class categorization groups in the British cohorts[7-10] ("High" = professional, managerial-technical; "Intermediate" = skilled non-manual; "Low" = non-skilled manual and partly or unskilled manual), and the Australian Standard Classification of Occupations in the Australian cohort ("High" = managers, administrators, professionals; "Intermediate" = tradespersons, clerical, service, sales; "Low" = production, transportation, elementary, labourer).[11] In the IPD-Work studies, occupational title was obtained from employers or some registers (COPSOQ-1,[15] COPSOQ-2,[16] FPS,[19] DWECS),[20] or from questionnaires completed by participants (Whitehall II,[13] WOLF-N,[14] WOLF-S).[15] In HeSSup,[18] SES was defined on the basis of a participant's self-reported highest educational qualification. As in previous studies,[12] the harmonised SES was categorised into low, intermediate, and high. Proportions of participants in each SES category by study are shown in **Table A1**.

We harmonised covariates: age, sex, smoking (never, ex- or current smoker), body mass index (BMI categories), physical activity (sedentary, moderate, highly active) and alcohol consumption (none, moderate, intermediate, heavy). In addition, we assessed shift working, a risk factor for type 2 diabetes.[21] Participants who reported daytime work only (that is, between 6:00 A.M. and 6.00 P.M.) were classified as non-shift workers, and those reporting night time work (between 6:00 P.M. and 6:00 A.M.) or any form of shift work were classified as shift workers.[22]

Definition of incident type 2 diabetes varied between the studies. In all open access studies,[1-11] diabetes was self-reported – disease at follow-up but absent at baseline defined incident cases. In IPD-Work studies,[13-20] the outcome was the first record of type 2 diabetes, diagnosed corresponding to ICD-10 code E11 (i.e., non-insulin-dependent diabetes mellitus including adult-onset, maturity-onset, nonketotic, stable, type II diabetes as well as non-insulin-dependent diabetes of the young, but excludes malnutrition-related, neonatal, pregnancy, childbirth and the puerperium diabetes, glycosuria, impaired glucose tolerance and postsurgical hypoinsulinaemia). We collected records from hospital admissions and discharge registers and mortality registers with a mention of diagnosis of type 2 diabetes in any of the diagnosis codes. Additionally, in the Finnish datasets (FPS,[19] HeSSup),[18] participants were also defined as an incident type 2 diabetes case the first time they appeared in the nationwide drug reimbursement register as eligible for type 2 diabetes medication. In the Whitehall II study,[13] type 2 diabetes was ascertained by 2-h oral glucose tolerance test administered every 5 years using World Health Organization criteria or by self-reports of diabetes diagnosis and medication.[23] The date of incident diabetes was defined as the date of the first record during the follow-up in any of the previously mentioned sources.

We excluded participants with prevalent (existing) type 1 or type 2 diabetes at baseline. In the open-access cohort studies,[111] prevalent diabetes at baseline was self-reported. In IPD-Work, prevalent diabetes was defined using information from any of the following: hospital records (all studies except for Whitehall II),[13] baseline medical assessment (Whitehall II),[13] self-report from the baseline questionnaire (Whitehall II,[13] WOLF S,[14] and WOLF N[15], FPS,[19] COPSOQ-I,[16] COPSOQ-II,[17] HeSSup,[18]) and/or drug reimbursement register (HeSSup,[18] FPS).[19]

Table A1. Study-specific distributions of socioeconomic status (SES)

Study	SES			Total N
	Low	Intermediate	High	
ACL[1]	630 (43)	357 (24)	493 (33)	1480
Alameda[2]	590 (26)	1068 (47)	632 (28)	2290
BCS1970[9]	1952 (30)	1393 (22)	3134 (48)	6479
BHPS[7]	6416 (42)	4216 (28)	4490 (30)	15122
HILDA[1]	1144 (23)	1509 (31)	2216 (46)	4869
MIDUS[4]	869 (29)	741 (25)	1344 (45)	2954
NCDS[8]	2617 (34)	1676 (22)	3414 (44)	7707
NHANES I[3]	4164 (48)	2499 (29)	2017 (23)	8680
UndSoc[10]	5651 (35)	3539 (22)	6972 (43)	16162
WLSG[5]	1542 (28)	1657 (30)	2353 (42)	5552
WLSS[6]	827 (29)	828 (29)	1211 (42)	2866
FPS[19]	7663 (18)	22833 (52)	13000 (30)	43496
HeSSup[18]	3647 (25)	8593 (59)	2369 (16)	14609
Whitehall II[13]	1130 (16)	3279 (45)	2854 (39)	7263
WOLF N[15]	2731 (60)	1481 (32)	359 (8)	4571
WOLF S[14]	1659 (31)	2841 (53)	880 (16)	5380
COPSOQ-I[16]	773 (44)	506 (29)	477 (27)	1756
COPSOQ-II[17]	1390 (42)	944 (29)	968 (29)	3302
DWECS[20]	2347 (43)	1643 (30)	1426 (26)	5416

Additional analyses

Subgroup analysis: The z-statistics was used to formally test the difference in the long working hours-diabetes association in the high and low SES groups. Z = [log(RR1) – log(RR2)] / sqrt(SE12 + SE22), where RR is the pooled estimate of the subgroup and SE is the standard error of the log(RR) estimate. The corresponding p-value was determined from the normal distribution.[23] The confidence intervals of the estimate in the low-SES group (risk ratio 1.29, 95% confidence interval 1.06-1.57) did not overlap the estimate in the high-SES group (risk ratio 1.00, 95% confidence interval 0.80-1.25), and vice versa. The z-statistic of 1.67 (P=0.048) indicated statistically significant difference in these estimates using a one-tailed test (P=0.048) and marginally significant (P=0.09) when using two-tailed test. The long working hours-diabetes association did not vary according to the method of diagnosing diabetes (z=0.11, P two-tailed=0.92), length of follow-up (z=0.16, P=0.87), study location (multiple comparisons, all z<0.16, P>0.53), sex (z=0.11, P=0.92), age group (z=1.43, P=0.15), or obesity status (z=1.50, P=0.13).

Effect size based on odds ratio versus hazard ratio: The open-access studies had self-reported incident diabetes without a precise date leading us to use logistic regression to calculate odds ratios and 95% confidence intervals for the association between working hours and incident type 2 diabetes in each study. In the IPD-Work studies, the date of diagnosis was available and the proportional hazards assumption was found not to be violated leading us to use Cox proportional hazards models to generate hazard ratios and 95% confidence intervals. Hazard ratio indicates the relative risk of event occurrence at follow-up in the exposure group compared to the reference group in participants who have not experienced the event at baseline. Given that probability of incident type 2 diabetes was low (between 1% and 11%, average 2.2%), odds ratios were considered as close approximations of relative risk. In meta-analysis, they were combined with hazard ratios, resulting in a common estimate of effect size.[24]

We ran further analysis to test whether results would differ between open-access studies reporting odds ratio[1-11] compared to IPD-Work studies reporting hazard ratio.[13-20] The associations between long working hours and incident diabetes were very similar in open-access (summary odds ratio 1.08, 95% CI 0.91-1.27) and IPD-Work (summary hazard ratio 1.07, 95% C 0.89-1.27) studies. Furthermore, the association remained unchanged when IPD-Work studies were analysed with logistic regression (summary odds ratio 1.06, 95% CI 0.88-1.27).”

Test of reverse causation bias: We found no support for reverse causation bias, that is, that undiagnosed diabetes symptoms before the diagnosis would make employees increase or reduce their working hours. The summary estimate was little changed after exclusion of the first 3 years of follow-up (this analysis was possible to perform in IPD-Work data only).[13-20] In low-SES occupations, minimally-adjusted summary relative risk was 1.43 (95%CI 0.95-2.15),

incidence difference 20 per 10,000 person-years. In high SES occupations, the corresponding figures were: relative risk 0.75 (95%CI 0.41-1.37), incidence difference -8 per 10,000 person-years.

Diabetes risk in part time workers: In analysis of type 2 diabetes risk in part time workers (those working less than 35 hours per week), the risk ratio compared to those working normal (35-45) hours was 0.86 (95%CI 0.78, 0.95, incidence difference –7 per 10,000 person years), suggesting that diabetes risk was slightly lower in part-time workers. Study-specific estimates are shown in **Figure A1**.

Test of bias due to shift working: To examine whether the association between long working hours and incident type 2 diabetes is attributable to the effects of shift work, we repeated the analysis after excluding shift workers from the sample. In studies including information on shift work,[7,9,11,13,18,19] the association remained after exclusion: age- and sex- adjusted hazard ratio in low-SES workers 1.82, 95%CI 1.10-3.01, incidence difference 38 per 10,000 person-years (hazard ratio before exclusion of shift workers 1.51, 95% CI: 1.12-2.03, incidence difference 23 per 10,000 person-years).

Figure A1. Random-effects meta-analysis of unpublished studies of the age and sex-adjusted association between long working hours and incident type 2 diabetes.

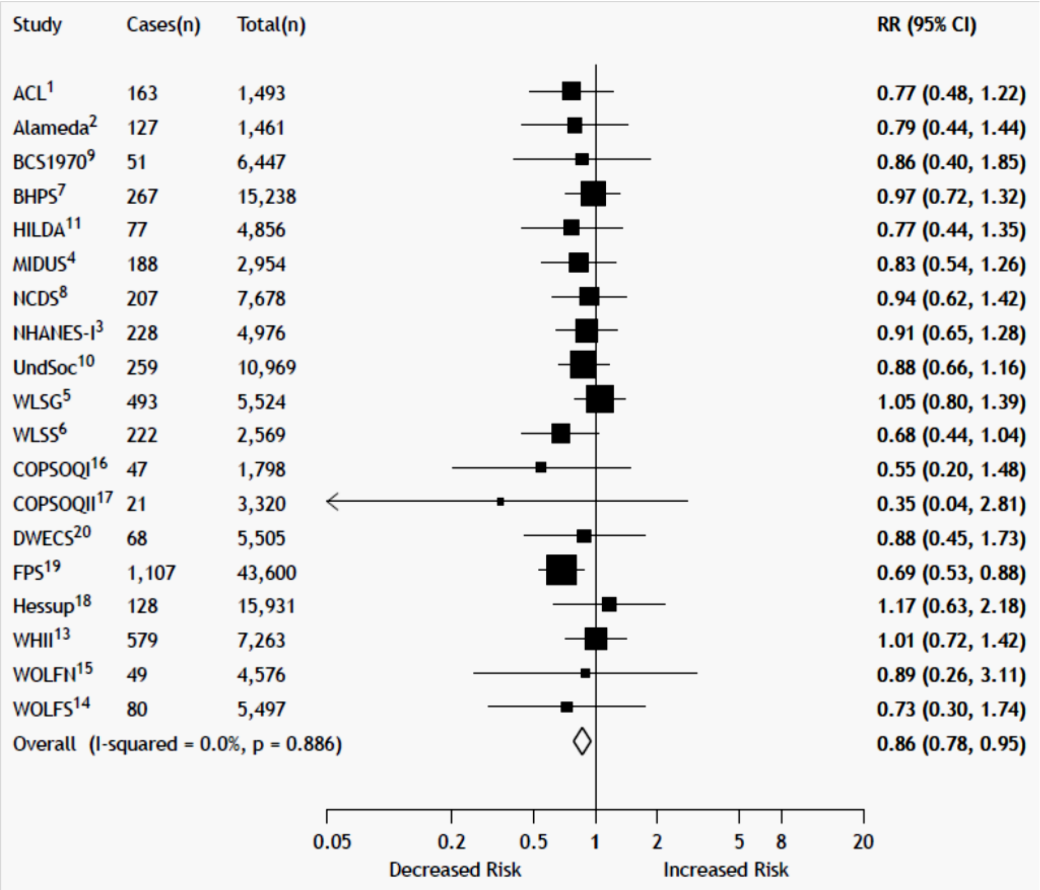

References

1. House JS, Lantz PM, Herd P. Continuity and change in the social stratification of aging and health over the life course: evidence from a nationally representative longitudinal study from 1986 to 2001/2002 (Americans' Changing Lives Study). *J Gerontol B Psychol Sci Soc Sci* 2005; **60** Spec No 2: 15-26.
2. Berkman L, Breslow L. Health and ways of living: the Alameda County Study. New York; 1983.
3. Madans JH, Kleinman JC, Cox CS, et al. 10 years after NHANES I: report of initial followup, 1982-84. *Public Health Rep* 1986; **101**: 465-73.
4. Brim OG, Ryff CD. How Healthy Are We? A National Study of Well-Being at Mid-Life. Chicago; 2004.
5. Sewell WH, Houser R. Education, occupation, and earnings: achievement in the early career. New York; 1975.
6. Hauser, Robert M. and William H. Sewell. Birth order and educational attainment in full sibships. *Am Educ Res J* 1985; **22**: 1-23.

7. Coxon APM. Sample design issues in a panel survey : The Case of the British Household Panel Study. Essex: Institute for Social and Economic Research; 1991.

8. Power C, Elliott J. Cohort profile: 1958 British birth cohort (National Child Development Study). *Int J Epidemiol* 2006; **35**: 34-41.

9. Elliott J, Shepherd P. Cohort profile: 1970 British Birth Cohort (BCS70). *Int J Epidemiol* 2006; **35**: 836-43.

10. McFall SL, Buck N. Understanding Society - the UK Household Longitudinal Survey: a resource for demographers. Netherlands; 2013.

11. Butterworth P, Crosier T. The validity of the SF-36 in an Australian National Household Survey: demonstrating the applicability of the Household Income and Labour Dynamics in Australia (HILDA) Survey to examination of health inequalities. *BMC Public Health* 2004; **4**: 44.

12. Kivimäki M, Nyberg ST, Batty GD, et al. Job strain as a risk factor for coronary heart disease: a collaborative meta-analysis of individual participant data. *Lancet* 2012; **380**: 1491-97.

13. Marmot MG, Davey Smith G, Stansfeld S, et al. Health inequalities among British civil servants: the Whitehall II study. *Lancet* 1991; **337**: 1387-93.

14. Peter R, Alfredsson L, Hammar N, Siegrist J, Theorell T, Westerholm P. High effort, low reward, and cardiovascular risk factors in employed Swedish men and women: baseline results from the WOLF Study. *J Epidemiol Community Health* 1998; **52**: 540-47.

15. Alfredsson L, Hammar N, Fransson E, et al. Job strain and major risk factors for coronary heart disease among employed males and females in a Swedish study on work, lipids and fibrinogen. *Scand J Work Environ Health* 2002; **28**: 238-48.

16. Kristensen TS, Hannerz H, Hogh A, Borg V. The Copenhagen Psychosocial Questionnaire--a tool for the assessment and improvement of the psychosocial work environment. *Scand J Work Environ Health* 2005; **31**: 438-49.

17. Pejtersen JH, Kristensen TS, Borg V, Bjorner JB. The second version of the Copenhagen Psychosocial Questionnaire. *Scand J Public Health* 2010; **38** (3 Suppl): 8-24.

18. Korkeila K, Suominen S, Ahvenainen J, et al. Non-response and related factors in a nation-wide health survey. *Eur J Epidemiol* 2001; **17**: 991-9.

19. Kivimäki M, Lawlor DA, Davey Smith G, et al. Socioeconomic position, co-occurrence of behavior-related risk factors, and coronary heart disease: the Finnish Public Sector study. *Am J Public Health* 2007; **97**: 874-79.

20. Feveile H, Olsen O, Burr H, Bach E. Danish Work Enviornment Cohort Study 2005: from idea to sampling design. *Stat Transit* 2007; **8**: 441-58.

21. Kivimäki M, Batty GD, Hublin C. Shift work as a risk factor for future type 2 diabetes: evidence, mechanisms, implications, and future research directions. *PLoS Med* 2011; **8**: e1001138.

22. Nyberg ST, Fransson EI, Heikkilä K, et al. Job strain as a risk factor for type 2 diabetes: A pooled analysis of 124 808 men and women. *Diabetes Care* 2014; **37**: 2268-75.

23. WHO. Definition, diagnosis and classification of diabetes mellitus and its complications Geneva: World Health Organization; 1997.

24. Borenstein M, Hedges LV, Higgins JPT, Rothstein HR. Introduction to meta-analysis. New York: Wiley, 2009.

25. Davies HT, Crombie IK, Tavakoli M. When can odds ratios mislead? *BMJ* 1998; **316**: 989-91.
